# Health-Promoting Properties of* Eucommia ulmoides*: A Review

**DOI:** 10.1155/2016/5202908

**Published:** 2016-03-02

**Authors:** Tarique Hussain, Bi'e Tan, Gang Liu, Oso Abimbola Oladele, Najma Rahu, M. C. Tossou, Yulong Yin

**Affiliations:** ^1^Observation and Experiment Station of Animal Nutrition and Feed Science in South-Central China, Ministry of Agriculture, Hunan Provincial Engineering Research Center for Healthy Livestock and Poultry Production, Key Laboratory of Agro-Ecological Processes in Subtropical Region, Institute of Subtropical Agriculture, Chinese Academy of Sciences, Changsha, Hunan 410125, China; ^2^University of the Chinese Academy of Sciences, Beijing 10008, China; ^3^Hunan Collaborative Innovation Center for Utilization of Botanical Functional Ingredients, Changsha, Hunan 410000, China; ^4^Department of Animal Nutrition, College of Animal Science and Livestock Production, Federal University of Agriculture, Abeokuta 110101, Nigeria; ^5^Department of Veterinary Microbiology, Faculty of Animal Husbandry and Veterinary Sciences, Sindh Agriculture University, Tando Jam, Sindh 70050, Pakistan

## Abstract

*Eucommia ulmoides* (EU) (also known as “Du Zhong” in Chinese language) is a plant containing various kinds of chemical constituents such as lignans, iridoids, phenolics, steroids, flavonoids, and other compounds. These constituents of EU possess various medicinal properties and have been used in Chinese Traditional Medicine (TCM) as a folk drink and functional food for several thousand years. EU has several pharmacological properties such as antioxidant, anti-inflammatory, antiallergic, antimicrobial, anticancer, antiaging, cardioprotective, and neuroprotective properties. Hence, it has been widely used solely or in combination with other compounds to treat cardiovascular and cerebrovascular diseases, sexual dysfunction, cancer, metabolic syndrome, and neurological diseases. This review paper summarizes the various active ingredients contained in EU and their health-promoting properties, thus serving as a reference material for the application of EU.

## 1. Introduction

*Eucommia ulmoides* (EU) (commonly called “Du Zhong” in Chinese language) belong to the family of Eucommiaceae, a genus of the small tree native to Central China [1]. This plant is widely cultivated in China on a large scale because of its medicinal importance. About 112 compounds have been isolated from EU which include lignans, iridoids, phenolics, steroids, and other compounds. Complementary herbs formula of this plant (such as delicious tea) has shown some medicinal properties. The leaf of EU has higher activity related to cortex, flower, and fruit [2, 3]. The leaves of EU have been reported to enhance bones strength and body muscles [4], thus leading to longevity and promoting fertility in humans [5]. Delicious tea formula made from the leaf of EU was reported to reduce fattiness and enhance energy metabolism. Flavonoid compounds (such as rutin, chlorogenic acid, ferulic acid, and caffeic acid) have been reported to exhibit antioxidants activity in the leaves of EU [6].

Although there has been enough literature on phytochemical properties of EU, few studies however existed on the pharmacological properties of the various compounds extracted from the barks, seeds, stems, and leaves of EU. This review paper will elucidate detailed information regarding different compounds extracted from the various parts (barks, seeds, stem, and leaf) of EU and the prospective uses of these compounds in health-promoting properties with scientific lines of evidence and thus provide a reference material for the application of EU.

## 2. Chemical Composition of* Eucommia ulmoides*

Various compounds isolated from different parts of EU are shown in Table 1.

### 2.1. Lignans and Iridoids

Lignans and their derivatives are the key components of EU [7]. To date, 28 lignans (such as bisepoxylignans, monoepoxylignans, neolignans, and sesquilignans) have been isolated from bark, leaves, and seeds of EU. Iridoid glycoside, a class of secondary metabolites, is the second main component of EU. Iridoids are typically found in plants known as glycosides. Twenty-four iridoids have been isolated and identified from EU (Table 1). These isolated compounds include geniposidic acid, aucubin, and asperuloside which have been reported to have wide pharmacological properties [8–10]. Two new compounds of iridoids, Eucommides-A and -C, have recently been isolated. These two natural compounds are considered as conjugates of iridoid and amino acids. However, the mechanism underlying their activity is not available [11].

### 2.2. Phenolic Compounds

Phenolic compounds which are derived from the foods have been reported to have positive impact on human health [12, 13]. About 29 phenolic compounds have been isolated and identified from EU [14]. Total content of phenolic compounds (in gallic acid equivalents of all the extracts) was analyzed using the Folin-Ciocalteu phenol reagent. Effects of seasonal variation on the contents of some compounds and antioxidants have been reported. Within the same year, higher contents of phenolics and flavonoids were discovered in the leaves of EU in August and May, respectively. Rutin, quercetin, geniposidic acid, and aucubin existed in higher concentration in May or June [15]. Moreover, higher activity of 1,1-diphenyl-2-picrylhydrazyl (DPPH) radical scavenging activity and metal ion chelating ability were found in the leaves of EU harvested in August. Increased content of food antioxidants was also reported in May when compared to other periods of the year [15]. The leaf of EU has been found to be a rich source of aminoacids, vitamins, minerals, and flavonoids such as quercetin, rutin, and geniposidic acid [11, 16]. A total of 7 flavonoids have been isolated from* Eucommia* plants [17]. Rutin and quercetin are the most important flavonoids [18]. Flavonoids are important compounds which are common in nature and are considered as secondary metabolites and function as chemical messengers, physiological regulators, and cell cycle inhibitors.

### 2.3. Steroids and Terpenoids

Six steroids and five terpenoids have been extracted and categorized from EU. These include *β*-sitosterol, daucosterol, ulmoprenol, betalin, betulic acid, ursolic acid, eucommidiol, rehmaglutin C, and 1,4*α*,5,7*α*-tetrahydro-7-hydroxymethyl-cyclopenta[c]pyran-4-carboxylic methyl ester which was specifically isolated from the bark of EU [19]. Loliolide has also been isolated from the leaves [20].

### 2.4. Polysaccharides

Polysaccharides from EU for 15 days at the concentrations of 300–600 mg/kg were reported to exhibit protective effects on kidneys as observed by malonaldehyde and glutathione levels after renal perfusions [21]. Histological examination also showed evidence of antioxidative properties. Extracts from the bark of EU using 70% ethanol also showed protective effects against cadmium at 125–500 mg/kg [22]. Histological examination also showed that EU in combination with* Panax pseudoginseng* at 25% and 50% weight, respectively, for six weeks at a dose rate of 35.7–41.6 mg/kg exerted light protective effects on glomerular filtration rate [8]. Two new polysaccharides have been separated from EU, which are eucomman A and B [23].

### 2.5. Other Ingredients and Chemicals

Amino acids, microelements, vitamins, and fatty acids have also been isolated from EU [17, 21–23]. Sun et al. also discovered new compounds such as n-octacosanoic acid, and tetracosanoic-2,3-dihydroxypropylester from EU [24].

Fatty acid composition of oil extracted from the seed of EU showed different concentrations of polyunsaturated fatty acids such as linoleic acid, linolenic acid (56.51% of total fatty acids, TFAs), and linolelaidic acid (12.66% of TFAs). Meanwhile, the main monounsaturated fatty acid isolated from the seed was found to be isoleic acid (15.80% of TFAs). Dominant saturated fatty acids isolated include palmitic acid and stearic acid which represent 9.82% and 2.59% of TFAs, respectively [25].

## 3. Health-Promoting Compounds of* Eucommia ulmoides*

### 3.1. Protective Effects on Cardiovascular System

In Chinese traditional medicines,* Eucommia* is considered as a major herbal tonic for cardiac patients.* Eucommia* bark extract is an active component used for antihypertensive formulations. It has been confirmed in many human as well as animal models as a vasorelaxant. Lignan from EU when administered to rats of the Okamoto strain (SHR) at the dose rate of 300 mg/kg for 16 weeks resulted in improved vascular remodeling and reduced mean arterial blood pressure. EU minimizes blood pressure at the dose of 500–1000 mg/kg. However, in high fructose fed diet, it develops insulin resistance and hypertension [26–28]. Supplementation of 500 and 1000 mg of EU for 8 weeks and thrice daily for 2 weeks showed minimal reduction in blood pressure and reduction in systolic and diastolic blood pressure [29]. Antihypertensive effect on the parasympathetic nervous system has been reported following the application of EU [30]. EU also serves as a vasorelaxative agent depending on nitric oxide and assumed to be linked with potassium channels [31]. EU has beta blocking potential which at 0.5% w/v reduces isoproterenol-stimulated lipolysis from 2.67 to 1.4 times the buffer control [29]. EU has been demonstrated to prevent hypertensive remodeling which is associated with aldose reductase inhibition [32]. The application of lignans from EU under condition of hypertension due to vascular remodeling was reported to serve as a new therapeutic agent [33].

EU also showed antihyperlipidemic properties by suppressing hepatic fatty acid and cholesterol biosynthesis [34]. In hyperlipidemic hamsters, dietary supplementation with leaf extract of EU at the dose of 0.175 g/100 g for 10 weeks reduced the concentrations of triglycerides, total cholesterol, low-density lipoprotein cholesterol (LDL-C), non-high-density lipoprotein cholesterol (non-HDL-C), and free acids in plasma and hepatic lipids compared to control group (fed 10 g, coconut oil, 0.2% cholesterol, w/w) [34]. In a similar manner, 1 mg or 5 mg intraduodenal injection of EU leaf extract reduced plasma triglyceride levels [65].

### 3.2. Antioxidant Effects

Antioxidant compounds from* Eucommia* plant reduced the level of free radicals [66, 67] and improved the disease condition caused by oxidative stress [68, 69]. Strong antioxidant properties of EU have been established under* in vivo* and* in vitro* studies [70, 71]. Extracts from EU reduced the level of hydrogen peroxide which expresses some caspase proteins by MC3T3E1 cells up to half concentration from 12.5 to 25 *μ*g/mL [71]. Extract of EU was reported to increase the actions of erythrocyte, superoxide dismutase, and catalase and glutathione peroxidase and reduce the concentration of hydrogen peroxide and lipid peroxide in erythrocytes, liver, and kidney [70]. Studies on diabetic rats indicated that superoxide dismutase (SOD) can be enhanced by* Eucommia* bark.* Eucommia* also increases the level of other antioxidant enzymes in the blood to neutralize free radicals [70].

Phenolics and flavonoids of medicinal herbs contributed significantly to oxidative activities in EU [34, 69–72]. Phenolics and flavonoids safely react with free radicals by donating a hydrogen atom or an electron and terminate chain reaction before the vital organs are damaged [73]. Antioxidant properties from leaves of the EU roasted cortex and seeds were analyzed by calculating radical scavenging activity of 2,2-diphenyl-1-picrylhydrazyl and ferric reducing antioxidant power and lipid peroxidation inhibition capacity in a *β*-carotene/linoleic acid system. Results indicated that leaf of the extract showed maximum DPPH radical scavenging activity with reducing rate and inhibition rate of 81.40%, followed by butylated hydroxytoluene (BHT) (76.60%) and the roasted cortex extract (16.72%). However, the seed extract had the lowest activity of 7.65%. In ferric reducing antioxidant power assays, the order of ferric reducing activities of EU extracts from leaf, seed, and roasted cortex was compared with positive control. In the *β*-carotene/linoleic acid emulsion system, the leaf extract showed better antioxidant capacity (43.58%) than the roasted cortex extract (26.71%) or seed extract (25.10%) [74].

In addition, aucubin compounds of EU have been demonstrated to exhibit photoprotective effects against oxidative stress. Ultraviolet (UV) B radiation produces free radicals in the skin which induce the synthesis of metalloproteinases (MMPs) causing photoaging in the skin, wrinkling, and discoloration which are prone to cancer. Aucubin played a vital role in defense mechanism against free radicals caused by UV irradiation [75].

### 3.3. Antibacterial, Antiviral, and Anti-Inflammatory Activity

EU have been reported to inhibit the growth of bacteria and reduce the secretion of proinflammatory cytokines in few studies. Ethanol extracts of EU at the dose rate of 0.1 and 1.0 mg/mL of 95% (v/v) were reported to exhibit some antibacterial (against* Acinetobacter baumannii* and* Staphylococcus aureus*) and antifungal (against* Aspergillus fumigatus*) effects [15–34, 65–76]. Furthermore, it has been reported that the same concentration of 0.1 mg/mL EU extracts reduces the secretion of proinflammatory cytokines including tumor necrosis factor-alpha (TNF-*α*), interleukin-8 (IL-8), and IL-1*β* by human monocytic (THP-1) cells pretreated with heat-killed* P. acnes*. Aqueous extract of EU significantly decreased cyclooxygenase-2 (COX-2) enzyme with IC_50_ = 9.92 mg/mL, although the effects were lowered compared with nonsteroid anti-inflammatory drugs [54]. Cortex of EU at the concentration of 0.1 and 0.5 mg/mL decreased production of (TNF-*α*, IL-6, and COX-2) prostaglandin E and nitric oxide [77].

Suppression of HIV infection has also been reported with daily intake of EU extracts or its alkaline extracts in tea formula. Alkaline extract of EU leaf in combination with 22% uronic acid, 27% reducing sugars, and 46% neutral sugars reduced HIV-induced cytopathicity (HTLV-III) with extremely low cytotoxicity in infected MT-4 cells (EC_50_) [78]. Lv et al. also demonstrated that the samples from EU Oliver had potent inhibitory activity against the HIV gp41 six-helix bundle formation [79].

### 3.4. Antiobesity Effects

Previous studies have shown that EU has antiobesity and antimetabolic syndrome properties [8, 26, 34, 80, 81]. It has been demonstrated that both* Eucommia* leaf extract (ELE) and* Eucommia* green leaf powder (EGLP) markedly suppressed body weight and white adipose tissue (WAT) in female ICR mice fed high-fat diets (HFD). The antiobesity effect of* Eucommia* green leaf extract (EGLE) has been linked to various compounds such as geniposidic acid, asperuloside, and chlorogenic acid which was isolated from the extract [8]. Application of water extract from the leaf of EU at the rate of 5% diet was reported to reduce fat accumulation rate in osteoporotic mice [4] although application of 500–1000 mg/kg EU leaf extract beyond 4 weeks showed no effect on fat accumulation in fructose overfed rats [26].

Antiobesity and antimetabolic syndrome activity in rat fed with a 35% high-fat diet could be maintained through secretion and regulation of adipocytokines that depend on the accumulation of visceral fat to improve insulin resistance or hyperlipidemia [80]. Administration of EU extracts at the concentration of 300–1600 mg/kg intake has been reported to enhance gene expressions for fat oxidation [81]. Administration of the extract was confirmed to increase fat oxidation in liver [34, 54, 65–81]. This increased fat oxidation in liver following administration of EU extract was attributed to the rate limiting stages of *β*-oxidation (CPT1A, ACOX1, and ACADVL), *α*-oxidation, and *ω*-oxidation (CYP4A1) [81].

### 3.5. Neuroprotective Effects

The stem bark extract of EU exhibited acetylcholinesterase inhibition properties* in vitro* (172 *μ*g/mL) IC_50_ and neuroprotective effects against beta-amyloid proteins [4]. It also inhibits 30–70% of cytotoxicity and efficacy of oxidative biomarkers when applied at a concentration of 2.5 *μ*g/mL [10]. Stem bark extract of EU showed higher protection activity against memory dysfunctions at the dose of 10–20 mg/kg with intracerebral injection of beta-amyloid proteins in rats [4].

### 3.6. Metabolic Modulation and Bones

*Eucommia* cortex extract can be used in the control of osteoporosis. This is because* Eucommia* extract is actively involved in mechanisms which initiate osteoblast, enhance osteogenesis, decrease osteoclast, and thus prevent osteolysis [82]. Total glycosides from* Eucommia ulmoides* seed (TGEUS) have been shown to improve bone density and femur strength in rats [83]. Daily administration of TGEUS at the rate of 400 mg/kg body weight/day to normal and Dawley rats was reported to significantly increase bone mineral density and showed improvements in microarchitecture structure of the femur bone [83].

*Eucommia* cortex extract was reported to induce the release of growth hormone (GH) responsible for bone maturation and bone remodeling. Products of alcoholic extraction from* Eucommia* bark were reported to be very potent in the release of growth hormone secretagogue. Increasing signals of estrogen receptor alpha has been shown to increase the growth of bone [84]. An exception to this effect was noticed in ovariectomized rats which showed no effect on the growth of bone [47, 82]. In menopausal research model, 5% diet of the EU was observed to minimize the bone loss in ovariectomized rats [61].* Eucommia* cortex fed at the dosage of 300–500 mg/kg showed reduced bone mass which is not significantly different from group fed with estradiol drug [61]. Antioxidant properties of* Eucommia* leaf extract were also reported to contribute positively to the promotion of bone growth by improving cell integrity during oxidative stress when applied at a reduced dosage (6.25 *μ*g/mL) [71]. Therefore,* Eucommia* extract can be established as a therapeutic agent under conditions of osteoporosis [85].

### 3.7. Phytoestrogenic Properties

EU was reported to exhibit phytoestrogenic and androgenic properties [84].* Eucommia* bark contains isoflavonoids, with estrogen like properties, which bind to human estrogen receptors. None of these isoflavonoids has male hormone like effect that interacts with human androgen receptor.* Eucommia* bark has been reported to show bimodal phytoandrogenic and hormone enhancing effects [84]. Androgen receptors play a key role in male as well as in female physiology such as skeletal muscle development, bone density, and sex drive [86, 87]. Ethanol extracts of* Eucommia* bark were reported to attach in a weak manner to activated androgen receptors with high affinity and produce testosterone at the rate of 5–25 ng/mL in mammalian COS-7 cells [84]. Oral induction of the ethanol extract showed no increase in prostatic weights at the dose of 1–50 mg. However, 20% increases in prostatic weights were observed by increasing the dosage up to 5000 *μ*g injection [84]. Application of EU at a concentration of 50 ng/mL enhanced the signals of estradiol in a manner similar to androgen receptors [84]. However, the promoting effect of EU on the cortisol and progesterone receptors was not observed [84].

*In vivo* animal studies conducted using oral administration of EU extracts potentiated androgenic and hormonal effects. A form of tripartite synergism between sex steroid receptors, sex hormones, and lipidic augmenters isolated from EU was found by Ong and Tan [84]. It has been shown that the activities of sex hormone in the body are optimized with the application of EU [84].

### 3.8. Hepatoprotective Effects

Study was conducted on different doses of* Eucommia ulmoides* and carbon tetrachloride on Sprague Dawley male rats to investigate the protective effects of EU in response to CCl_4_ induced acute liver lipid accumulation. Results demonstrated that* Eucommia ulmoides* Oliv. cortex extracts (EUCE) significantly decreased the hepatic lipid accumulation induced by CCl_4_. EU enhances lysosomal enzyme activity relieving protein folding requirement which turns into attenuation of ER stress. ApoB secretion was improved by effects of ER stress; along this, it regulates biotransformation of CCl_4_ and its resultant inhibition of ROS accumulation [88].

## 4. Future Perspective and Conclusion

This review paper discusses health-promoting properties of EU on cardiovascular system and antioxidant, antibacterial, antiviral, anti-inflammatory, antiobesity, and neuroprotective effects and metabolic modulation on bones and phytoestrogenic properties. These health-promoting properties have attracted much interest in the extraction and functional development of active ingredients of EU. In further studies, molecular mechanisms underlying certain health-promoting properties of EU need to be explored.

## Figures and Tables

**Table 1:** Compounds isolated from various parts of *Eucommia ulmoides*.

Category	Compounds	References
Bark of *Eucommia ulmoides*		

Lignans	(+)-1-Hydroxypinoresinol-4′,4′′-di-O-*β*-D-glucopyranoside	[35]
	(+)-1-Hydroxypinoresinol-4′-O-*β*-D-glucopyranoside	[36]
	(+)-1-Hydroxypinoresinol-4′′-O-*β*-D-glucopyranoside	[36]
	(+)-Epipinoresinol	[37]
	(+)-l-Hydroxypinoresinol	[37]
	(+)-Medioresinol	[37]
	(+)-Medioresinol-di-O-*β*-D-glucopyranoside	[38]
	(+)-Pinoresinol	[37]
	(+)-Pinoresinol-4′-O-*β*-D-glucopyranoside	[38]
	(+)-Pinoresinol-di-O-*β*-D-glucopyranoside	[39]
	(+)-Pinoresinol-4-O-*β*-D-glucopyranosyl(1–6)-*β*-D-glucopyranoside	[40]
	(+)-Syringaresinol	[37]
	(+)-Syringaresinol-O-*β*-D-glucopyranoside	[35]
	Eucommin A (+)-medioresinol-4′-*β*-D-glucopyranoside	[35]
	Liriodendrin (+)-syringaresinol-di-O-*β*-D-glucopyranoside	[38]

Phenolics	Astragalin	[11, 41]
	Isoquercetin	[11, 42]
	Quercetin	[41]
	Quercetin-3-O-galactoside (hyperin)	[41]
	Quercetin-3-O-xyloglucoside	[42]
	Rutin	[11–24]
	Wogonside	[43]
	(−)-Epieateehin	[24]
	(±)-Threo-guaiacyl glycerol	[36]
	Caffeic acid	[44]
	Catechin	[24]
	Chlorogenic acid	[11, 24]
	Coniferol	[37]
	Erythro-guaiacylglycerol-*β*-coniferyl aldehyde ether	[37]
	Eucophenoside	[45]
	Methyl chlorogenate	[37]
	Protocatechuic acid	[46]
	Threo-guaiacylglycerol-*β*-coniferyl aldehyde ether	[37]
	Vanillic acid	[47]

Iridoids	Deoxyeucommiol	[48]
	Eucommiol-II	[48]
	Eucommiside-I	[48]
	Genipin	[37]
	Geniposide	[48]
	Geniposidic acid	[11, 48]

Monoepoxylignans	(−)-Olivil-4′,4′′-di-O-*β*-D-glucopyranoside	[35]
	(+)-Cycloolivil	[36]
	(+)-Olivil	[36]
	(+)-Olivil-4′-O-*β*-D-glucopyranoside	[36, 49]
	(+)-Olivil-4′′-O-*β*-D-glucopyranoside	[36]

Neolignans	Citrusin B	[50]
	Dehydrodiconiferylalcohol-4,*γ*′-di-O-*β*-D-glucopyranoside	[50]
	Dihydroxydehydrodiconiferyl alcohol	[37]
	Erythro-dihydroxydehydrodiconiferyl alcohol	[37]
	Threo-dihydroxydehydrodiconiferyl alcohol	[37]

Sesquilignans	(−)-HedyotolC-4′,4′′′-di-O-*β*-D-glucopyranoside	[48]
	Syringylglycerol-*β*-syringaresinol ether-4′′-4′′′-O-*β*-D-glucopyranoside	[50]
	Syringylglycerol-*β*-syringaresinol ether-4′′-4′′′-O-*β*-D-glucopyranoside	[50]

Steroid and terpenoid	1,4*α*,5,7*α*-Tetrahydro-7-hydroxymethyl-cyclopenta[c]pyran-4-carboxylicmethyl ester	[19]
	Betalin	[47]
	Betulic acid	[47]
	Daucosterol	[51]
	Eucommidiol	[19]
	Rehmaglutin C	[19]
	Ursolic acid	[47]

Others	(*α*R)-*α*,4,2′,4′-Tetrahydroxydihydrochalcone	[43]
	(*α*R)-*α*-O-*β*-D-Glucopyranosyl-4,2′,4′-trihydroxydihyd	[43]
	4,2′,4′-Trihydroxychalcone	[43]
	Eucomman A	[52]
	Eucomman B	[23]
	n-Oetaeosanoic acid	[24]
	Quercetin-3-O-*α*-L-arabinopyranosyl-(1-2)-*β*-D-glucopyranoside	[53, 54]
	Tetraeosanoie-2,3-dihydroxypropyl ester	[24]

Leaves of *Eucommia ulmoides*		

Phenolics	Astragalin	[11, 41]
	Hirsutin	[17]
	Isoquercetin	[11, 42]
	Kaempferol	[17]
	Kaempferol-3-O-6′′-acetyl-glucoside	[55]
	Kaempferol-3-O-rutinoside	[11]
	Quercetin-3-O-*α*-L-arabinopyranosyl-(1-2)-*β*-D-glucopyranoside	[53, 54, 56]
	Rutin	[11, 24]
	Ajugoside	[57]
	Asperuloside	[11]
	Asperulosidic acid	[58]
	Aueubin or aueuboside	[57]
	Deacetyl asperulosidic acid	[58]
	Eucommiol	[57]
	Eucommioside	[59]
	Eucomoside A	[11]
	Eucomoside B	[11]
	Eucomoside C	[11]
	Geniposidic acid	[11, 36]
	Harpagide acetate	[57]
	Reptoside	[57]
	Scandoside-10-O-acetate	[11]
	Ulmoside	[59, 60]
	3-(3,4-Dihydroxyphenyl) propionic acid	[60]
	3(3-Hydroxyphenyl-propionic acid)	[60]
	3,4-Dihydrobenzonic acid	[17]
	Caffeic acid	[37, 61]
	Chlorogenic acid methylester	[62]
	Eatechol	[60]
	Isochlorogenic acid A	[42]
	Isochlorogenic acid C	[42]
	p-trans-Coumaric acid	[58]
	Pyrogallol	[58]

Monoepoxylignans	(+)-Olivil-4′-O-*β*-D-glucopyranoside	[36, 49]

Steroid and terpenoid	Loliolide	[18]
	Ulmoidol	[63]

Others	Ethyl glucopyranoside	[21]

Seeds of *Eucommia ulmoides*		

Iridoids	Ulmoidoside A	[64]
	Ulmoidoside B	[64]
	Ulmoidoside C	[64]
	Ulmoidoside D	[64]

Phenolics	Dihydrocaffeic acid	[64]

Stems of *Eucommia ulmoides*		

Phenolics	Coniferin	[44]
	Koaburaside	[44]
	Syringin	[44]
